# Integrated multi-omics analysis and microbial recombinant protein system reveal hydroxylation and glycosylation involving nevadensin biosynthesis in *Lysionotus pauciflorus*

**DOI:** 10.1186/s12934-022-01921-2

**Published:** 2022-09-19

**Authors:** Tianze Wu, Li Xiang, Ranran Gao, Lan Wu, Gang Deng, Wenting Wang, Yongping Zhang, Bo Wang, Liang Shen, Shilin Chen, Xia Liu, Qinggang Yin

**Affiliations:** 1 School of Chemistry Chemical Engineering and Life Sciences, Wuhan University of Technology, No. 122, Lo Lion Road, Wuhan, 430070 Hubei China; 2 Key Laboratory of Beijing for Identification and Safety Evaluation of Chinese Medicine, Institute of Chinese Materia Medica, China Academy of Chinese Medical Sciences, Beijing, 100700 China; 3 Artemisinin Research Center, China Academy of Chinese Medical Sciences, Beijing, 100700 China; 4 College of Pharmaceutical Sciences, National Engineering Technology Research Center for Miao Medicine, Guizhou University of Traditional Chinese Medicine, Guiyang, 550025 Guizhou China; 5 Beijing Museum of Natural History, Beijing Academy of Science and Technology, Beijing, 100050 China

**Keywords:** Nevadensin, Flavone, CYP450, Glycosyltransferase, Microbe, *Lysinotus pauciflorus*

## Abstract

**Background:**

Karst-adapted plant, *Lysionotus pauciflours* accumulates special secondary metabolites with a wide range of pharmacological effects for surviving in drought and high salty areas, while researchers focused more on their environmental adaptations and evolutions. Nevadensin (5,7-dihydroxy-6,8,4'-trimethoxyflavone), the main active component in *L. pauciflours,* has unique bioactivity of such as anti-inflammatory, anti-tubercular, and anti-tumor or cancer. Complex decoration of nevadensin, such as hydroxylation and glycosylation of the flavone skeleton determines its diversity and biological activities. The lack of omics data limits the exploration of accumulation mode and biosynthetic pathway. Herein, we integrated transcriptomics, metabolomics, and microbial recombinant protein system to reveal hydroxylation and glycosylation involving nevadensin biosynthesis in *L. pauciflours.*

**Results:**

Up to 275 flavonoids were found to exist in *L. pauciflorus* by UPLC-MS/MS based on widely targeted metabolome analysis. The special flavone nevadensin (5,7-dihydroxy-6,8,4'-trimethoxyflavone) is enriched in different tissues, as are its related glycosides. The flavonoid biosynthesis pathway was drawn based on differential transcripts analysis, including 9 PAL, 5 C4H, 8 4CL, 6 CHS, 3 CHI, 1 FNSII, and over 20 OMTs.

Total 310 LpCYP450s were classified into 9 clans, 36 families, and 35 subfamilies, with 56% being A-type CYP450s by phylogenetic evolutionary analysis. According to the phylogenetic tree with AtUGTs, 187 LpUGTs clustered into 14 evolutionary groups (A-N), with 74% being E, A, D, G, and K groups.

Two LpCYP82D members and LpUGT95 were functionally identified in *Saccharomyces cerevisiae* and *Escherichia coli*, respectively*.* CYP82D-8 and CYP82D-1 specially hydroxylate the 6- or 8-position of A ring in vivo and in vitro, dislike the function of F6H or F8H discovered in basil which functioned depending on A-ring substituted methoxy. These results refreshed the starting mode that apigenin can be firstly hydroxylated on A ring in nevadensin biosynthesis. Furthermore, LpUGT95 clustered into the 7-OGT family was verified to catalyze 7-O glucosylation of nevadensin accompanied with weak nevadensin 5-O glucosylation function, firstly revealed glycosylation modification of flavones with completely substituted A-ring.

**Conclusions:**

Metabolomic and full-length transcriptomic association analysis unveiled the accumulation mode and biosynthetic pathway of the secondary metabolites in the karst-adapted plant *L. pauciflorus*. Moreover, functional identification of two LpCYP82D members and one LpUGT in microbe reconstructed the pathway of nevadensin biosynthesis.

**Supplementary Information:**

The online version contains supplementary material available at 10.1186/s12934-022-01921-2.

## Background

*Lysionotus pauciflorus*, a typical karst-adapted plant, is used in the treatment of lymph node tuberculosis, cough with tachypnoea, and rheumatic pains [1, 2]. For containing some special secondary metabolites, *L. pauciflorus* grows companying with anti-diverse abiotic stresses such as soil salinity, drought, and some other extreme environmental conditions [3, 4]. Nevadensin (5,7-dihydroxy-6,8,4'-trimethoxyflavone), a main active component in the plant, has already established itself as a promising natural bioactive substance that bears the potential to become a novel “natural lead” in drug discovery programmes, including hypotensive, anti-inflammatory, anti-tubercular, anti-tumor or cancer, and anti-microbial activities [5]. Complex decoration of nevadensin such as hydroxylation and glycosylation toward the flavone skeleton determines its diversity and biological activities [4].

Hydroxylation in nevadensin biosynthesis in *L. pauciflorus* enhances its high pharmacological efficacies, which mainly be contributed by cytochrome P450 monooxygenases (CYP450s) [6]. The hydroxylation toward the 6- or 8-position at the A-ring of flavone is catalyzed by flavone 6-hydroxylase (F6H) or flavone 8-hydroxylase (F8H), respectively. CYP71D9 (F6H) from *Glycine max* catalyzed the conversion of flavanones more efficiently than flavones and was demonstrated to be responsible for the hydroxylation at the 6-position of A-ring of flavonol [7]. Other CYP82 family members in Lamiaceae were identified as having similar functions for the A-ring of flavone. CYP82D1.1 (*Scutellaria baicalensis*), CYP82D33 (*Ocimum basilicum*), and CYP82D62 (*Mentha* x *piperita*) were responsible for the hydroxylation at position 6 of ring A. Interestingly, MpCYP82D62 catalyzed the 6-hydroxylation of genkwanin and showed the same substrate requirements (7-methoxy, 5-hydroxy residues) as its homolog ObCYP82D33, while SbCYP82D1.1 has broad substrate specificity for flavones such as chrysin and apigenin and is responsible for the biosynthesis of baicalein and scutellarein in roots and aerial parts of *S. baicalensis*, respectively [6, 8]. The purified recombinant ObF8H-1 belonging to the pheophorbide a oxygenase (PAO)-like subfamily of Rieske-type oxygenases displayed high affinity for salvigenin and was inactive with other tested flavones except for cirsimaritin, which was 8-hydroxylated with less than 0.2% of the reaction rate with salvigenin as substrate, whereas SbCYP82D2 was an F8H with high substrate specificity, accepting only chrysin as its substrate to produce norwogonin, although minor 6-hydroxylation activity can also be detected [8, 9]. Subsequently, Gao et al. identified seven *Scutellaria*-specific CYP82D genes encoding F8H, predicting the function of F6H evolved from F8H by multi-collinearity and phylogenetic analysis of CYP82D in *Scutellaria* [10]. The catalytic characteristics of the CYP82D family in Lamiaceae are differentiated, therefore the functions of this family from *L. pauciflorus* belonging to Gesneriaceae are still a mystery.

Nevadensin is most commonly conjugated with sugar moieties by UDP-dependent glycosyltransferases (UGTs) which could efficiently increase its soluble ability, such as nevadensin 5-O-β-D-glucoside, nevadensin 7-O-β-D-glucoside and nevadensin 7-O-[α-L-rhamnosyl (1-6)]-β-D-glucoside. It’s not hard to see that the O-glycosylation only occurred at the 5-OH or 7-OH position in A ring, while other hydrogen positions were replaced by methoxy in nevadensin. Based on the proposed pathway of nevadensin in basil, we hypothesize that the glycosyltransferases in *L. pauciflorus* were specially forced to handle adding glycosyl to 5-OH or 7-OH after the methylation at 6-OH and 8-OH in A ring finished. UGTs possess the substrate regioselectivity rather than substrate specificity as other enzymes, usually clustered to 3-OGTs (3-O-glucosyltransferases), 5-OGTs, 7-OGTs, and branch-forming cluster phylogenetic tree analysis [11]. Therefore, many LpUGTs could be predicted as 5-OGTs or 7-OGTs for the special structure of nevadensin glycosides. Both CYP450 and UGT are superfamilies, and the lack of gene sequences directly limits the phylogenetic analysis and further gene function exploration.

Recent advances in genome sequencing and various omics technologies have greatly facilitated the discovery of candidate genes involved in metabolite biosynthesis and improved the understanding of metabolite biosynthesis molecular mechanisms. A transcriptomic assessment of *Aralia elata* allowed researchers to identify 150 CYP450s and 92 UGTs, of which 4 CYP450s and 5 UGTs were proposed to play roles in triterpenoid saponin biosynthesis [12]. Similarly, one prior RNA-seq analysis of three *Panax notoginseng* plants led the authors to identify 350 and 342 predicted unigenes encoding CYP450s and UGTs, respectively [13]. During multi-omics periods, microbial heterologous expression is wildly used to identify key gene functions in the flavonoid biosynthesis pathway, especially in un-model plants. Based on big omics data, Yin et al. identified the glycosylation of rutin in tartary buckwheat, panasenoside in ginseng, and flavonol glucoside in horse chestnut using prokaryotic expression system [14–16]; Gao et al. and Zhao et al. identified the hydroxylation of *Scutellaria*-specific flavone in *S. cerevisiae*, respectively [8, 10].

Herein, up to 275 flavonoids were found to exist in *L. pauciflorus* by widely targeted metabolome analysis. 96,277 transcripts were obtained by analyzing the transcriptomic profiles of four different tissues of *L. pauciflorus* performing single-molecule real-time sequencing (SMRT) and next-generation sequencing (NGS). Combining multi-omics analysis and synthetic biology tools, key genes involved in the flavone biosynthesis pathway were identified, especially systematic analysis of CYP450 and UGT family members. Subsequently, candidate CYP450s and UGTs were verified to catalyze the 6- or 8-hydroxylation and 5- and 7-glycosylation in nevadensin biosynthesis using *S. cerevisiae* and *E. coli*. Decoding of decorated enzymes would benefit to further elucidate the entire biosynthetic pathway for nevadensin, and these identified genes could be used to modify flavone or produce nevadensin glycosides in microbes.

## Results

### Flavone glycosides were abundant in *L. pauciflorus* based on metabolome analysis

**Fig. 1:**
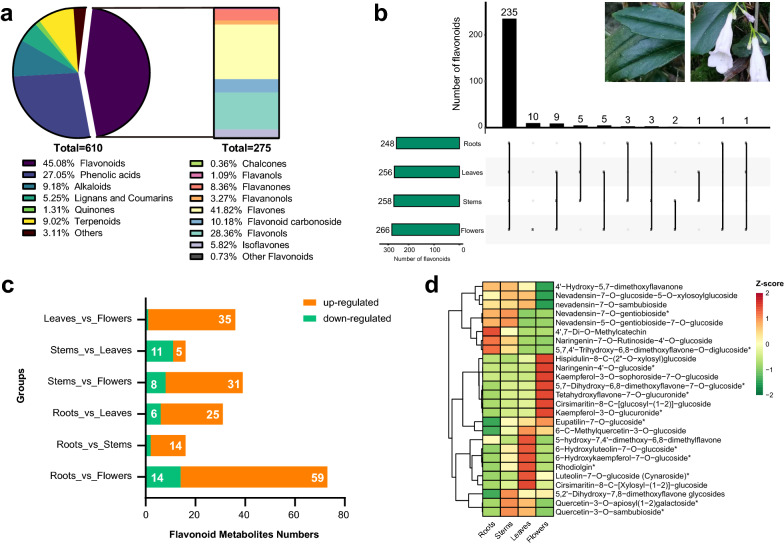
Widely targeted metabolome analysis of *L. pauciflorus*. **a** The classification of all metabolites and flavonoids identified. **b** Analysis of the flavonoid metabolite types from four tissues of *L. pauciflorus*. The dot means the flavonoids detected in tissues on the right. Black rectangles represent the number of flavonoids common to tissues labeled by the dot below. Green rectangles represent the number of flavonoids in one tissue. **c** The differential analysis of flavonoid metabolite content between two tissues. **d** Heatmap of 26 flavonoids selected from the top 10 flavonoid metabolites in each tissue. Red indicates the high content of the flavonoids and green indicates the low content of the flavonoids

To investigate the accumulation mode in roots, stems, leaves, and flowers of *L. pauciflorus* surviving in a unique environment, the secondary metabolites were identified by UPLC-MS/MS analysis. A total of 610 putative metabolites were detected and could be categorized into seven primary classes, including 275 flavonoids, 165 phenolic acids, 56 alkaloids, 32 lignans and coumarins, 55 terpenoids, 8 quinones, and 19 others (Fig. 1a). Flavonoids (45.08%) were the main metabolites which were identified 248, 256, 258 and 266 from the roots, stems, leaves, and flowers, respectively. The four tissues shared 235 flavonoids (Fig. 1b). Flowers almost included all 275 flavonoids, and 10 flavonoids were only identified in it, including nevadensin-5-O-malonylglucoside, isoquercitrin, tricin-7-O-(2''-O-glucosyl) glucoside, isorhoifolin, 5,7,3',4'-tetrahydroxy-6-methoxyflavone-8-C-[glucosyl-(1–2)]-glucoside, hispidulin-8-C-glucoside, 5-hydroxy-6,7,3',4'-tetramethoxyflavanone, isorhamnetin-3-O-rhamnoside, diosmetin-7-O-(6''-malonyl)glucoside, and apigenin-6-C-(2''-glucuronyl)glucoside. Pairwise comparisons indicated that 14 and 59 flavonoids were down- and up-regulated in flowers compared to the roots, respectively. Meanwhile, 35 and 31 flavonoids were up-regulated in flowers compared to the leaves and stems, respectively (Fig. 1c), indicating that flowers might be the best tissue for further study of flavonoid biosynthesis in *L. pauciflorus* among four tissues. Moreover, a total of 26 flavonoids was selected to investigate the accumulation pattern in *planta*, from the top 10 flavonoids in each tissue. We found that kaempferol glycosides were accumulated in flowers, while 4 nevadensin glycosides were more abundant in roots, stems, and leaves. (Fig. 1d and Additional file 1: Table S1).

### Expression patterns of genes involving nevadensin biosynthesis in *L. pauciflorus*

To further explore the pathway of nevadensin biosynthesis in *L. pauciflorus*, the expression patterns of genes related to nevadensin biosynthesis were analyzed. Four different tissues (root, stem, leaf, and flower) were subjected to paired-end sequencing using the HiSeq 6000 platform, with 706,750,886 reads produced. Meanwhile, full-length cDNAs from twelve pooled poly (A) RNA samples were normalized and subjected to SMRT sequencing using the PacBio platform, generating a total of 333,787,309,810 raw reads. After filtering and assembly, 95,938 unigenes and 96,277 transcripts were generated with a 2050 bp N50 (Additional file 2: Table S2). A large number of unigenes and transcripts could drive further elucidation of the nevadensin biosynthesis pathway.

**Fig. 2:**
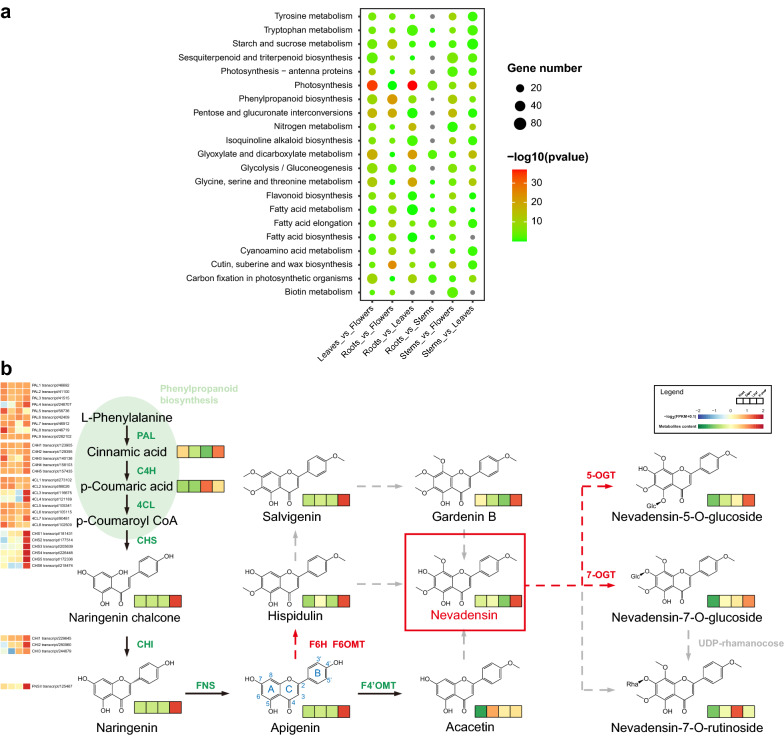
The KEGG analysis based on the transcriptome and putative biosynthesis pathway for nevadensin in *L. pauciflorus*. **a** Comparative analysis of the KEGG functional enrichment of differentially expressed genes (DEGs) in different groups. **b** The proposed pathway of nevadensin in *L. pauciflorus*. The heatmap highlights the patterns of expression for these genes in root, stem, leaf, and flower, with FPKM values used for normalization and red-to-blue color-coded. The relative contents of flavones in four tissues are in red-to-green color-coded. Arrow code: black solid line, evidence this conversion occurs in the plant; red dotted line, biochemically favorable step; grey dotted line, no current information as to whether or how the reaction occurs. Color code: green, confirmed enzyme; red, enzyme relevant to this study. Flavonoid backbone numbering and ring nomenclature are indicated on the apigenin structure in blue coded. Nevadensin was in a red line rectangle. F6/8H, flavone 6/8 hydroxylase; F6/8OMT, flavone 6/8-O methyltransferase; 5/7-OGT, 5/7-O Glycosyltransferase

These transcripts were annotated with the GO, KEGG, KOG, Nr, Pfam, Swiss-Prot, and TrEMBL, leading to the base function annotation of 79,383 (85%) transcripts. Besides, special function databases including CARD, CAZy, PHI, and VFDB were used to annotate, leading to the annotation of 10,955 (11%) transcripts (Additional file 3: Table S3). The KEGG enrichment analysis revealed that most genes were mainly enriched in “Photosynthesis” (72 in group leaves vs flowers), “Phenylpropanoid biosynthesis” (58 in group roots vs flowers), “Pentose and glucuronate interconversions” (50 in group leaves vs flowers) and “Glycolysis / Gluconeogenesis” (68 in group leaves vs flowers) for flowers vs leaves, roots, stems (Fig. 2a). Combined the results of KEGG annotation of transcriptome and BLASTP, 32 transcripts were found to be associated with the phenylpropanoid and flavonoid biosynthesis, including 9 transcripts encoding phenylalanine ammonia lyase (PAL), 5 transcripts encoding cinnamate 4-monooxygenase (C4H), 8 transcripts encoding 4-coumarate-CoA ligase (4CL), 6 transcripts encoding chalcone synthase (CHS), 3 transcripts encoding chalcone isomerase (CHI) and 1 transcript encoding flavone synthase (FNS). Subsequently, the putative pathway involved in the phenylpropanoid and flavonoid biosynthesis was drawn combined with cinnamic acid, *p*-cinnamic acid, naringenin chalcone, naringenin, apigenin, acacetin, hispidulin, salvigenin, gardenin B, nevadensin and nevadensin 5/7-O-glycoside in metabolome (Fig. 2b). Predictably, the expression levels of CHS, CHI and FNS were highest in flowers, consistent with the accumulation mode of naringenin chalcone, naringenin and apigenin in tissues.

According to the structure of nevadensin and its glycoside compounds, there were three main decorate steps: hydroxylation at positions 6 and 8 of A-ring mainly by CYP450s, multiple regiospecific O-methylation by O-methyltransferases (OMT) and O-glycosylation by O-glycosyltransferase (OGT). Thus, the hypothetical pathway to nevadensin in *L. pauciflorus* was shown in Fig. 2b based on the present evidence of transcriptome, metabolome, and significant decoration of apigenin. The tissue-specific flavone distribution patterns were supposed to be a valuable reference for identifying CYP450s and UGTs involved in the downstream biosynthesis pathway of nevadensin in *L. pauciflorus*.

### Phylogenetic analysis of LpCYP450s and functional identification of LpF6/8H in yeast

**Fig. 3:**
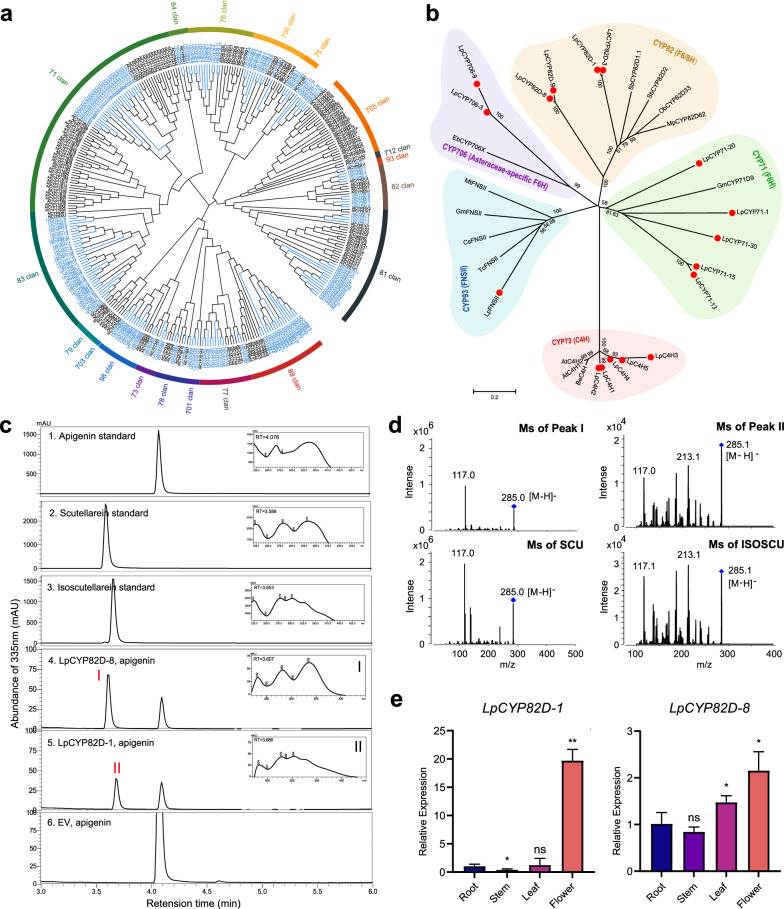
Identification of LpF6/8H by phylogenetic analysis and enzymatic tests in yeast. **a** Phylogenetic tree of A-type (71 clans) CYP450s in *L. pauciflorus* and *Arabidopsis thaliana*. The neighbor-joining tree was constructed using Mega 11.0 with 1000 bootstrap replicates. The respective protein names and numbers are listed in (Additional file 11: Table S6). The CYP450 members from *L. pauciflorus* are marked in red. **b** Phylogenetic tree of previously characterized flavonoid biosynthesis CYP450s and those LpCYP450s isolated in this study (red circle). **c, d** Identification of the enzymatic products of recombinant LpCYP82D-8 and LpCYP82D-1 proteins by UPLC and UPLC/MS/MS. The peaks marked in red were the products of in vivo yeast reaction. SCU: Scutellarein; ISOSCU: Isoscutellarein. **e** Relative expression of two LpCYP82D genes in different tissues determined by qRT-PCR

According to the common nomenclature system conducted by David R. Nelson, total 310 LpCYP450 genes were classified into 9 clans, 36 families, and 35 subfamilies, with 56% being A-type CYP450s and 44% being non-A-type CYP450s (Additional file 4: Figure S1a and Additional file 5: Table S4) [17]. The CYP71 clan was shown in Fig. 3a, containing 175 genes belonging to 15 families (CYP81, CYP82, CYP93, CYP75, CYP706, CYP76, CYP84, CYP71, CYP83, CYP98, CYP73, CYP78, CYP701, CYP77, CYP89). Previous studies revealed that F6H genes are all belonging to 71 clans (CYP71, CYP82, and Asteraceae-specific CYP706 families) [6–8, 10]. To further explore the functional roles of LpCYP450s, the expression profiles of A-type LpCYP450s in four tissues were conducted (Additional file 4: Figure S1b). Integrated the phylogenetic evolutionary analysis and expression pattern in tissues (FPKM value > 60), ten LpCYP450 genes were selected and cloned to further functional study, including 5 CYP71, 1 CYP73, 4 CYP82D (Fig. 3b).

LpCYP82D-1 and LpCYP82D-8 were finally identified as F8H and F6H in yeast in vivo, respectively. The putative substrate including 4',5,7-trihydroxyflavone (apigenin), 5,7-dihydroxy-4'-methoxyflavone (acacetin), 5,7,4'-trihydroxy-8-methoxyflavone (4'-hydroxywogonin), 4',5,7-Trihydroxy-6-methoxyflavone (hispidulin), 8-hydroxyapigenin (isoscutellarein) or 4',5,6,7-tetrahydroxyflavanone (scutellarein) was added to the medium of WAT11 strains expressing *LpCYP82D-8* or *LpCYP82D-1* respectively. A large new peak I was detected in extracts from yeast expressing *LpCYP82D-8* adding apigenin (Fig. 3c trace 3). This new product had a retention time identical to that of the scutellarein standard Furthermore, peak I had the same tandem MS (MS/MS) pattern as the scutellarein standard, with fragments of m/z 285.0 and 117.0 (Fig. 3d). Peak II was detected in extracts from yeast expressing *LpCYP82D-1* adding apigenin (Fig. 3c trace4). The retention time of this new product is identical to that of the isoscutellarein standard. Peak II had the same tandem MS (MS/MS) pattern as the isoscutellarein standard, with fragments of m/z 285.1, 213.1, and 117.1(Fig. 3d). Moreover, there was a new peak detected in extracts from yeast expressing *LpCYP82D-8* or -1 adding acacetin, and the new product was predicted as hydroxyl acacetin through MS spectrum (m/z of pair ions: 299.1 and 284.1). A new product was found only in the extracts from the yeast with *LpCYP82D-1* adding 4′-hydroxywogonin, which was identified as 6, 4′-dihydroxywogonin by MS spectrum (m/z of pair ions: 181.0, 300.1 and 315.1) (Additional file 6: Figure S2a-b). Furthermore, to evaluate the catalyzing activity of LpCYP82Ds, the conversion ratio was calculated. The conversion ratio of LpCYP82D-8 was twice higher as that of SbCYP82D1.1 treated with the same substrate, whereas the activity of LpCYP82D-8 was significantly higher when treated with apigenin vs acacetin (Additional file 6: Figure S2c).

To test the in vitro function of the recombinant LpCYP82D-8 or -1 protein, extracted microsomal proteins containing LpCYP82D-8 or -1 were initially incubated with NADPH as a cofactor and the flavones mentioned above as the potential substrates. The results revealed that the recombinant LpCYP82D-8 or -1 had a similar function in vivo (Additional file 7: figure S3). Consequently, the LpCYP82D-8 enzyme was identified as an F6H, and the LpCYP82D-1 enzyme was identified as an F8H in vivo and in vitro.

To explore the potential molecular basis for the high enzymatic activity of LpCYP82D-8 towards apigenin, we analyzed the interactions among amino acid residues, the substrates (apigenin, acacetin, hispidulin, 4’-hydroxywogonin), and ferriporphyrin. We found that the selected substrates were all docked into the active pocket of LpCYP82D-1 or -8 (Additional file 8: Figure S4 and Additional file 9: Figure S5), worth mentioning that four substrates were almost overlapped with ferriporphyrin in active domains of LpCYP82D-8 docking models. The optimal binding free energy among the interaction of LpCYP82D-1, apigenin, and ferriporphyrin is -8.0 kcal/mol. Five amino acids (Arg113, Trp138, Arg390, Ala455, and Gly454) interacted with ferriporphyrin by H-bond, and three amino acids (Arg113, Ser130, and Arg458) could potentially form H-bonds with apigenin. However, more active residues were found in the docking result of LpCYP82D-8 with apigenin and ferriporphyrin (about eight active residues), and the optimal binding free energy (−8.1 kcal/mol) was lower. Six amino acids (Arg131, Ser389, His392, Ser458, Arg461, and Phe456) interacted with ferriporphyrin by H-bond, and four amino acids (Thr325, Ser389, Pro455, and Phe456) could potentially form H-bond with apigenin. Gao et reported that Asp113, Tyr115, Ser122, Ala319, Ile 327 or Leu 464 residues might be a contributing factor to the recruitment of F6H in SbCYP82D1, and its optimal binding free energy was −7.6 kcal/mol [10]. The more active residues and lower binding free energy of LpCYP82D-8 docking with apigenin than that of SbCYP82D1 were consistent with the enzymatic results.

To investigate the transcripts of *LpCYP82D-8* and *LpCYP82D-1* in *planta* via qRT-PCR, both of these LpCYP82D genes were expressed at higher levels in flowers (Fig. 3e), which were consistent with the tendency of FPKM in transcriptomic data (Additional file 10: Table S5).

### Phylogenetic analysis of LpUGTs and functional identification of Lp7-OGT in *E. coli*

**Fig. 4:**
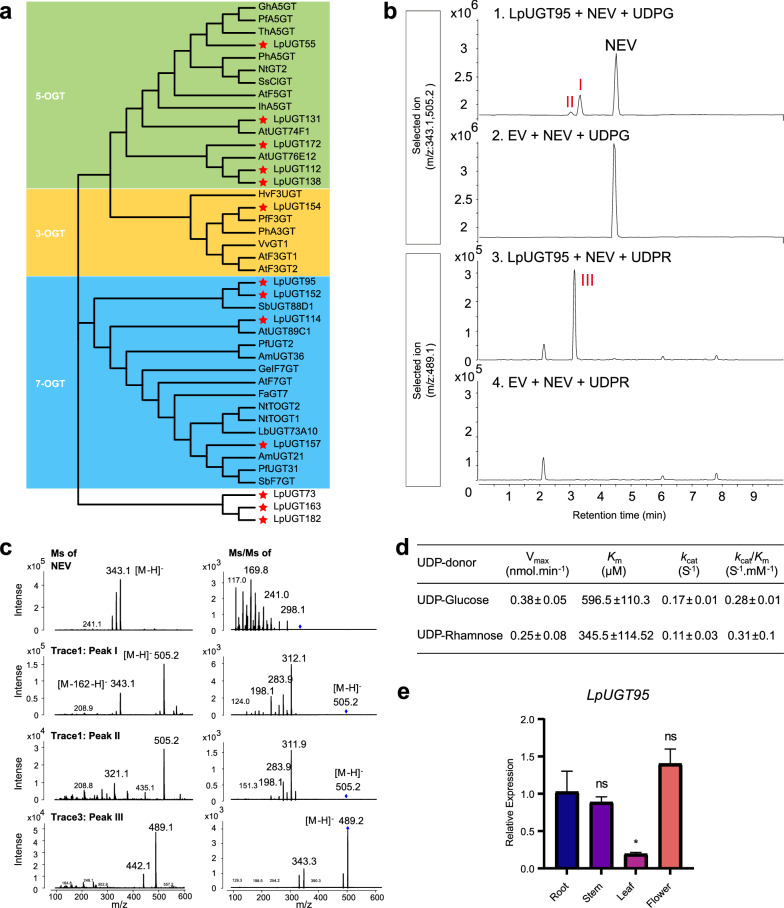
Regioselectivity prediction and identification of LpUGTs. **a** Phylogenetic tree of previously characterized flavonoid biosynthesis UGTs and those LpUGTs isolated in this study (red star). The functional proteins are listed in (Additional file 14: Table S7). The scale indicates 0.2 amino acid substitutions per site. **b**, **c** Identification of the enzymatic product of recombinant LpUGT95 protein by UPLC/MS/MS. EV, empty vector; UDPG, UDP-glucose; UDPR, UDP-rhamnose. **d** Kinetic parameters of the recombinant LpUGT95 proteins with nevadensin as acceptor substrate and UDP-glucose or UDP-rhamnose as donor substrate. Values are the average of three independent replicates; error bars represent the average ± one standard deviation. **e** Relative expression of *LpUGT95* in different tissues determined by qRT-PCR

A total of 187 transcripts were filtered with the criteria: have significant PSPG motifs near their C-terminals, and proteins encoded by these genes are comprised of 300–600 amino acids. The phylogenetic tree with 187 LpUGTs and 107 AtUGTs classified UGT families (Additional file 5: Table S4 and Additional file 11: Table S6). According to the phylogenetic tree, LpUGTs clustered into 14 evolutionary groups (A-N), and five groups among them cover 74% of LpUGTs: E (40 members), A (30), D (29), G (21), and K (19). After comparing the FPKM (> 60) and expression levels of all genes in four tissues, thirteen LpUGT genes were selected and constructed a phylogenetic tree with previously characterized UGT genes, including LpUGT154, 5 UGTs, and 4 UGTs belonged to 3-OGT, 5-OGT and 7-OGT cluster, respectively (Additional file 5: Table S4 and Additional file 12: Figure S6, and Fig. 4a). Their motifs were predicted by MEME v.5.4.1 (https://meme-suite.org/meme/tools/meme), including PSPG-box as motif 1 (marked as a red rectangle) (Additional file 13: Figure S7a). Nine LpUGT genes (*LpUGT55, LpUGT131, LpUGT172, LpUGT112, LpUGT138, LpUGT95, LpUGT152, LpUGT114* and *LpUGT157*) were finally selected as candidate genes encoding 5/7-OGT. Five LpUGTs out of nine candidates were cloned and expressed in *E. coli* to produce recombinant proteins.

Enzymatic tests were carried out by using 4 flavonoids (hispidulin, apigenin, acacetin, and nevadensin) as sugar acceptors, and using UDP-glucose or UDP-rhamnose as sugar donors. The results revealed that recombinant LpUGT95 could specifically use nevadensin as substrate, and it could use UDP-glucose or UDP-rhamnose as a sugar donor (Fig. 4b). Both peak I and II were detected in extracts from recombinant LpUGT95 treated with UDP-glucose (Fig. 4b trace1-2). The tandem MS (MS/MS) pattern of peak I contained highly intense fragments of m/z 343.1 (the same as the relative molecular mass of substrate nevadensin[M-H]-) and 505.2 (the same as the relative molecular mass of nevadensin-5/7-O-glucoside[M-H]-), and it was predicted as nevadensin 7-O glucoside. The tandem MS (MS/MS) pattern of peak II only contained fragments of m/z 505.2 (Fig. 4c). To ensure the structure of the product (peak II), MS/MS pattern of peak I, peak II and nevadensin standard were gained. Peak II had the same tandem MS/MS pattern as peak I, with fragments of m/z 311.9, 283.9, and 198.1, indicating that the product at peak II had the same mother ion as the product at the peak I. Peak III was detected in extracts from recombinant LpUGT95 treated with UDP- rhamnose (Fig. 4b trace3-4). The tandem MS (MS/MS) pattern of peak III only contained fragments of m/z 489.2 (the same as the relative molecular mass of nevadensin-O-rhamnoside [M-H]-) as well, while the tandem MS/MS pattern contained fragments of 489.2 and 343.3 (Fig. 4c). Therefore, LpUGT95 enzyme could be identified as a nevadensin 5/7-O glycosyltransferases.

Moreover, we found that the enzymatic efficiency of LpUGT95 toward nevadensin using UDP-glucose (*k*_cat_/*K*_m_ value, 0.28 S^−1^ mM^−1^) as sugar donor was similar to that of UDP-rhamnose (0.31 S^−1^ mM^−1^), though the affinity of LpUGT95 with nevadensin using UDP-rhamnose (*K*_m_ value, 345.5 μM) was almost twice than that of UDP-glucose (596.5 μM) (Fig. 4d). To explore the transcript of *LpUGT95* in *planta*, the expression profiles in four tissues were quantified via qRT-PCR. *LpUGT95* was expressed at lower levels in leaves (Fig. 4e), which was in the same tendency of FPKM in transcriptomes of different tissues (Additional file 10: Table S5).

## Discussion

### The flavone biosynthesis pathway in karst-adapted plant *L. pauciflorus* decoded by multi-omic analysis

The plants from Gesneriaceae are used to study the adaptation to the high salinity and drought stress in limestone karst, while less focus is on the special secondary metabolites [19, 20]. Combined NGS and SMRT sequencing, we obtained the full-length sequence of 96,277 transcripts and focused on the flavone pathway including CHS, CHI, FNS, OMT, CYP450s, and UGTs, especially predicting the process of nevadensin glycosides formation. High-quality gene sequence information makes it possible to analyze the phylogenetic evolution of superfamilies, such as CYP450s and UGTs. 310 CYP450s and 187 UGTs were divided into 9 clans and 14 evolutionary groups, respectively. Besides the groups we focused on, such as CYP71 and CYP82D families in the CYP450, or 5-OGT and 7-OGTs clusters in UGT, other genes could be used to explore terpenoid or alkaloids in *L. pauciflorus* metabolome listed in supplementary materials.

### Pathway of nevadensin biosynthesis reconstructed by LpF6/8H

**Fig. 5:**
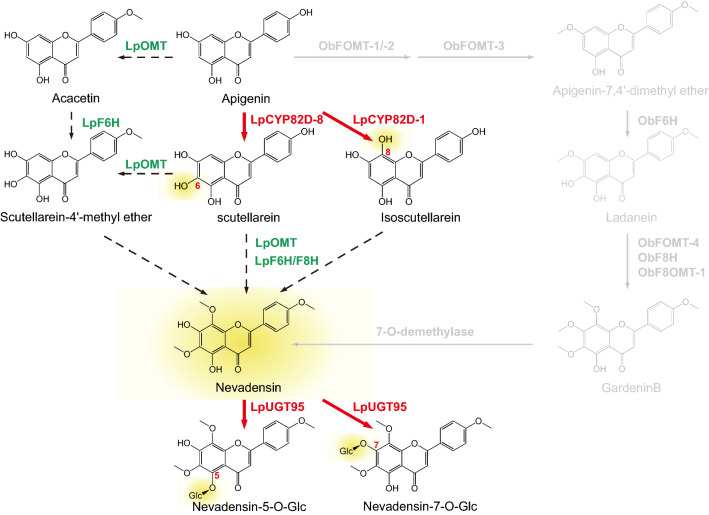
Modification of nevadensin catalyzed by characterized LpCYP82Ds (F6H/F8H) and LpUGT95 (7/5-OGT) compared with Berim’s study [18]. Line legend: the solid line represents the verified reaction in this study; the dotted line means the possible reaction which has not been verified. Color legend: The part of the pathway characterized in sweet basil was in grey; the enzymes verified in this study were in red; the possible reactions and corresponding catalytic enzymes were in black line and green font, respectively

Gang group had revealed the hydroxylation occurred before methylation in nevadensin biosynthesis using crude enzymes from trichomes in sweet basil. ObCYP82D33, MpCYP82D62, and ObF8H all could specially hydroxylate hydroxylation flavone of which A-ring substituted methoxyl residues, such as salvigenin and gardenin B. Subsequently, a unique 2-oxoglutarate-dependent flavone 7-O-demethylase uncovered the blind box of the hydroxylation and methylation of flavone in Basil (the grey part of Fig. 5) [9]. However, Zhao et al. and Gao et al. identified the CYP82D family possesses F6H and F8H toward 4'-deoxyflavone in *S. baicalensis*, respectively [8, 10]. Multi-collinearity and phylogenetic analysis of the CYP82D family in the *Scutellaria* genus confirmed that the function of F6H evolved from F8H. Our results identified four CYP82D members from full-length transcripts of *L. pauciflorus*, and two among them contributed to F6H and F8H function. Different from ObCYP82D33 and MpCYP82D62, LpCYP82D-8 (F6H) could hydroxylate the flavone with or without methoxyl residues (apigenin, acacetin, or 4′-hydroxywogonin) (Additional file 6: Figure S2 and Additional file 7: Figure S3). LpF6H located before SbCYP82D2, ObCYP82D33, and MpCYP82D62 in the phylogenetic tree, indicating that the function of F6H might occur before F8H in these species from Lamiales, contrary to their evolution in *Scutellaria* genus. LpCYP82D-1 (F8H) shared a similar function of 8-hydroxylation with ObF8H coming from the Rieske-type PAO family, though preferring to apigenin and no activity toward flavone with A-ring substituted methoxyl residues (4′-hydroxywogonin). Therefore, the discovery of LpF6H and LpF8H reconstructs the beginning of the pathway for navedensin biosynthesis in *L. pauciflorus*, which is different from sweet basil (Fig. 5).

### LpUGTs with substrate specificity and regioselectivity

The substrate specificity of UGTs contains glycosylate certain kinds of substrate and regioselectivity. Toward the special structure of nevadensin glycosides, the UGTs were predicted to transfer oriented glucoside to 5-OH or 7-OH of A ring, consistent with the results from our enzymatic tests. LpUGT95 was clustered into 7-OGTs by phylogenetic analysis; consistently, the products of the recombinant enzyme assay mainly were nevadensin 7-O-glucoside. Interestingly, we found the identified LpUGT have strong specificity to navedensin and no activity to other flavones tested. Whether UGT or OMT will first work in plants has been pending for they are all cytoplastic proteins. Our results indicated that glycosylation was conducted after methylation in *L. pauliflorus*, verifying our hypothesis. Meanwhile, substrate specificity and regioselectivity of LpUGTs must be determined by their structure, thus we would focus on the docking between the LpUGTs structure model and nevadensin to find key residues. From Fig. 5, the function of LpUG95 filled the gap of the final decoration on nevadensin. Unfortunately, the second glycosylation was not identified because our candidates were outside the branch-forming group. Unexpectedly, we found the products of LpUGT95 could rhamnosylate nevadensin, though its rhamnoside did not exist in our metabolome data; these new products could be candidates for drug discovery.

### The application prospects of identified CYP450s and UGTs

Drug discovery always needs several target compounds for in vivo or in vitro pharmacological tests. Only 3 species were developed to extract nevadensin during the reported 25 species containing the compound [5]. Moreover, a special growth environment limits the high production of nevadensin through planting. As a green manufacturing production mode, microbe-transformed new genes could provide a schedule for the high production of expected substances. Precisely speaking, new gene function is the core factor for key compounds biosynthesis. Antimalarial artemisinin biosynthesis relied on the discovery of ADH, CYP716AV1, and DBR2; the identification of CsPT4 and THCAS, and CBDAS pushed complete biosynthesis of cannabinoids [21, 22]. Berim et al. had realized the production of 8- and/or 6-substituted methoxylated flavones from their natural precursor apigenin in five strains of *S. cerevisiae* transformed with a set of genes from *O. basilicum* [23]*.* Besides complementing the nevadensin biosynthesis pathway, LpF6H, LpF8H or LpUGT95 identified here could be used as modules to produce different flavones or their glycosides in the microbe.

## Conclusions

The entire biosynthesis pathway of flavone was annotated combining full-length sequence and metabolome of *L. pauciflorus*. The superfamilies CYP450s and UGTs were systematically analyzed by phylogenetic trees. Identification of F6/8H and 5-/7-OGT in *S. cerevisiae* and *E. coli* revealed the special hydroxylation and glycosylation for nevadensin, respectively.

## Methods

### Plant materials and chemicals

*L. pauciflorus* samples were collected from Anlong County, Southwest Guizhou Autonomous Prefecture, China. The roots, stems, leaves, and flowers were cut into small pieces and rapidly frozen in liquid nitrogen and then stored at 80℃ for further metabolic and transcriptomic analysis. All experiments were analyzed with three biological repeats.

The substrates tested in the present study were purchased from Wuhan Jonk Biological Technology Co., Ltd, including nevadensin, 4'-hydroxywogonin, hispidulin, isoscutellarein, scutellarein, acacetin, and apigenin. NADPH, UDP-glucose, and UDP-rhamnose were purchased from Solarblo, Sigma-Aldrich (Oakville, CA, USA), and Xian Qiyue Biological Technology Co., Ltd, respectively. The chemicals used in this study were all of analytical or HPLC grade.

### Total metabolite profiling in *L. pauciflorus*

Freeze-dried samples (100 mg) in a vacuum freeze drier were ground using a bead beater (30 Hz, 1.5 min, three repetitions, MM400, Restsch). The samples were suspended in 70% methanol (1200 μL), kept at 4 °C overnight, vortexed thrice during the extraction, and centrifuged at 12,000 g for 10 min at 4 °C. The supernatant was eventually filtered through a 0.22 μm pore size mesh, then used for UPLC-MS/MS.

The data acquisition instrument system mainly included ultra-performance liquid chromatography (UPLC) (SHIMADZU Nexera X2, https://www.shimadzu.com.cn/) and tandem mass spectrometry (MS/MS) (Applied Biosystem 4500 QTRAP, http://www.appliedbiosystems.com.cn/). Liquid-phase conditions were as follows: (i) chromatographic column: Agilent SB-C18 1.8 μm, 2.1 mm × 100 mm; (ii) mobile phase: the aqueous phase was ultrapure water (0.1% acetic acid was added), whereas the organic phase was acetonitrile (0.1% acetic acid was added); (iii) elution gradient: 0 min water/acetonitrile (95:5 v/v), 5:95 v/v at 9.0 min, 5:95 v/v at 10.0 min, 95:5 v/v at 11.1 min, and 95:5 v/v at 14.0 min; and (iv) flow rate: 0.35 mL/min; the column temperature was 40 °C; the injection quantity was 4 μL.

### RNA extraction, next-generation sequencing, and SMRT sequencing

The total RNA of the above samples was extracted according to the manual instructions in the RNeasy plant mini kit (Qiagen, Germany), and then the quality was examined. Complementary DNA (cDNA) libraries were constructed by Benagen Biotechnology Co., Ltd (Wuhan, China) based on sequencing using the synthesis technology, then sequenced using the Illumina HiSeq6000 and PacBio Sequel platform. The raw NGS data were filtered using the FastQC program (http://www.bioinformat ics.babraham.ac.uk/projects/fastqc/). Raw SMRT sequencing reads were filtered using SMRT Link v10.1. Full-length transcript (FLNC) sequences were acquired using the Iso-Seq process. Any redundancy in high-quality full-length transcripts was removed by CD-HIT. To ensure the accuracy and validity of transcript results, transcript results were evaluated from different aspects using N50, ExN50, and BUSCO (91.3%). The gene expression level was quantified by fragments per kilobase of transcript per million mapped reads (FPKM) which could normalize the number of reads and the length of transcripts calculated by RSEM v1.3.1 [24, 25]. Analyses for differential expression between intergroup samples were conducted by DESeq2. R package (version 1.12.3), setting parameters with fold Change ≥ 5 and P-value < 0.05 [26].

### LpCYP450 and UGT genes identification and phylogenetic analysis

The known CYP450 and UGT protein sequences (PF00067, CYP450; PF00201, UGT) from the Pfam database (https://pfam.xfam.org/) were downloaded and used as a query in Hidden Markov Model (HMM) searches for candidate genes in *L. pauciflorus*, with the cutoff at 0.01 [27]. Moreover, all CYP450 and UGT protein sequences in *Arabidopsis* were downloaded from TAIR (https://www.arabidopsis.org/index.jsp), which were applied as a query to blast the protein sequences in *L. pauciflorus* transcriptome database. Subsequently, the HMMER and BlastP results were merged, and deleted redundant sequences. To confirm the domain organization, the candidate protein sequences were characterized using CDD (https://www.ncbi.nlm.nih.gov/Structure/bwrpsb/bwrpsb.cgi), and the sequences lacking conserved domain were eliminated manually. In total, 310 CYP450s (175 A-type, 135 non-A-type) and 187 UGTs were analyzed further (Additional file 5: Table S4). Multiple sequence alignment was conducted using ClustalX v2.1 software with the default parameters. The phylogenetic dendrograms were constructed using the MEGA v11 program based on the alignment of the protein sequences with the Neighbor-joining (NJ) method [28]. A total of 1000 bootstrap replicates were computed. EvolView (http://www.evolgenius.info/evolview/#/) was used to optimize the evolutionary tree.

### Isolation of LpCYP450s and LpUGTs

Primers for the 10 LpCYP450 and 5 LpUGT genes were designed according to SMRT coding sequences of *L. pauciflorus*. All the forward and reverse primers (Additional file 15: Table S8) for gene cloning contained corresponding restriction sites. Mixed cDNAs from roots, stems, leaves, and flowers of *L. pauciflorus* were used for gene amplification. The PCR products were purified and digested using the corresponding restriction enzymes. The PCR products of LpCYP450s and LpUGTs were then ligated to the pESC-HIS and pMAL-c2x vector (New England BioLabs, Ipswich, MA, USA) digested with the same restriction enzymes for expression of recombinant protein in yeast and *E. coli*, respectively.

### Enzyme assay and product identification

For the eukaryotic expression system, gaining engineered yeast strains recombinant CYP proteins was followed as previously described [10]. The yeast expression vector pESC-HIS constructs with *LpCYP450s* or an empty vector were transformed into yeast *S. cerevisiae* WAT11, an engineered strain overexpressing an *Arabidopsis* NADPH cytochrome P450 reductase gene [29]. The recombinant yeast strains were first grown in 20 ml SD-His medium containing 20 g L^−1^ glucose at 30 ℃ for 24 h. To ensure complete digestion of glucose, 200 μL of strain solution was used with urine glucose test strips. After the glucose test, the yeast cells were harvested by centrifugation at 1000 g for 5 min and washed twice with ddH2O. SD-His medium with 20 g L^−1^ galactose was used to resuspend the strains. 1 mL strains were used for yeast in vivo reaction. The substrate apigenin, acacetin, hispidulin, 4’-hydroxywogonin, scutellarein, or isoscutellarein was supplemented at 100 μM into the cultures. After 16 h, ethyl acetate was used to stop the reaction, and the supernatant was concentrated at low pressure after ultrasonic centrifugation until ethyl acetate was completely volatilized, and the pellet was dissolved in 200 μL methanol and stored at 4 ℃ for further UPLC analysis. Microsomal proteins were isolated according to the protocol described by Truan and were dissolved in protein storage buffer (20% [v/v] glycerol, 100 mM Tris-HCl [pH 7.5]) [30]. Protein concentrations were determined using Bradford’s assay [31]. The flavone hydroxylases were assayed in 100 μL of reaction volume, which contained 35 mM Tris–HCl (pH 7.4), 1 mM dithiothreitol, about 2.0 μg of crude protein extract, 50 μM substrate, and 1 mM NADPH. The assays were incubated for 1 h at 30℃. The conversion ratios of LpCYP82s were calculated by the peak area of the product divided by the sum of the peak area of the product and substrate. SbCYP82D1.1 was used as a positive control. Each reaction has three replicates.

For the prokaryotic expression system, recombinant UGT proteins were gained following as previously described [14]; the crude proteins were used for the enzymatic activity test. The substrate specificity of LpUGT95 was analyzed with 100 μM candidate flavones at 37 ℃ for 30 min. The recombinant LpUGT95 protein was purified for further experiments. For kinetic analysis of LpUGT95 protein, 10 μg purified enzymes were incubated in reaction mixtures comprising 10 mM DTT, 50 mM Tris-HCl (pH = 7.4), and 2 mM UDP-glucose or UDP-rhamnose, in a final volume of 50 μL. The concentration of the tested acceptor substrates ranged from 1 to 400 μM. Methanol was added to stop reactions after 30 min incubation at 30 ℃. Samples were centrifuged at 14,000 rpm for 5 min and stored at 4 ℃ for further UPLC analysis. The kinetic parameters *K*_m_ and *k*_cat_ were calculated by using the Hyper 32 program (http://hyper32.software.informer.com/).

The enzymatic reaction solution was filtered through 0.22 μm membranes. 2 μl aliquot was used to analyze the new products by ultra-high performance liquid chromatography (UPLC, Nexera UPLC LC-20A system, SHIMADZU, Japan). The UPLC separation conditions for flavonoids are as follows: Agilent InfinityLab Poroshell 120 EC-C18 column (2.1*50 mm 1.9-micron), water containing 0.1% formic acid as mobile phase A, acetonitrile as mobile phase B (0 − 7 min, 5–100% B; 7–9 min, 100% B; 9–10.5 min, 100-5% B; 10.5–11.5 min, 5%B), flow rate 0.3 mL/min, UV detector (335 nm). 1 μl aliquot was injected into UPLC-MS/MS for the analysis of products. An Agilent 1290 Infinity II HPLC coupled with an Agilent QTOF 6530 instrument (ion source, ESI) and QQQ-MS (Agilent 6470) were used as previously described [16]. Samples were eluted on an Agilent Infinity Lab Poroshell 120 EC-C18 column (2.1*50 mm 1.9-micron). We used water containing 0.1% formic acid and acetonitrile as mobile phase solvents A and B, respectively. Flavone glucosides were eluted using a linear gradient program: 0 min, 5% B; 7 min, 95% B; 1 min for washing. The MS conditions fixed for negative-ion mode were as follows: a negative ion mode, the gas temperature at 300 °C, gas flow at 6 L min^−1^, nebulizer at 30 psi, sheath gas temperature at 300 °C, sheath gas flow at 11 L min^−1^, the capillary voltage at 2500 V, and nozzle voltage at 2000 V. We detected metabolites in the PI mode, auto MS/MS mode and targeted MS/MS mode; 30 V collision energy was used to obtain ion fragments.

### Homology modeling and molecular docking

Homology models of LpCYP450s were built using the crystal structures of SmCYP76AH1 (*Salvia miltiorrhiza*, PDB No., 5YWA) as templates on the Phyre2 server at http://www.sbg.bio.ic.ac.uk/phyre2. The identity of LpCYP82D-8 or -1 with SmCYP76AH1 is 25%. The ferriporphyrin and four substrates were respectively docked with the model structure of CYP82D-8 and -1 using AutoDock Vina [32]. The molecular graphics were rendered with PyMOL 2.5 (http://www.pymol.org).

### Expression analysis by quantitative real-time PCR

Total RNA was isolated from roots, stems, leaves, and flowers of *L. pauciflorus* by using an RNAprep Pure Plant Kit (Tiangen Biotech Co., Beijing, China). The *LpActin* was used as the housekeeping. gene in qRT-PCRs which was selected by blasting with *AtACT2*. PCR conditions were as follows: 5 ℃ for 2 min, 9 ℃ for 10 min, then 40 cycles of 9 ℃ for 10 s, 6 ℃ for 15 s, and 72 ℃ 20 s. Primer sequences used for qRT-PCR were listed in Additional file 15: Table S8.

### Statistical analysis

Statistical analyses were performed using Prism v9.0. P-values were calculated using an unpaired, two-legged Student’s t-test (**p < 0.01; *p < 0.05; ns, not significant). Data represent means ± standard deviation (n ≥ 3).

## Supplementary Information

**Additional file 1: Table S1.** The relative content of flavonoids at four tissues of *L. pauciflorus.*

**Additional file 2: Table S2.** Summary of the RNA-seq and full-length non-chimeric (FLNC) analysis of *L. pauciflorus.*

**Additional file 3: Table S3. **The number of functional genes annotated to different annotation databases based on SMRT data.

**Additional file 4: Figure S1.** Identification of CYP450s in *L. pauciflorus*. **(a)** Phylogenetic evolutionary analysis of non-A-type CYP450s from *L. pauciflorus*. (**b**) Hierarchical clustering for expression profiles of 175 A-type LpCYP450s.

**Additional file 5: Table S4.** Summary of identified *LpCYP450s* and *LpUGTs.*

**Additional file 6: Figure S2. **The identification of LpCYP82D-8 and -1 function *in vivo* by UPLC and MS/MS, and the conversion ratio of LpCYP82D-8. (**a**) UPLC profile of LpCYP82D-8 and -1 treated with different substrates. (**b**) MS/MS results of yeast expressing LpCYP82D-8 and -1 added acacetin standard as substrate. (**c**) The conversion ratio of LpCYP82D-8 treated with apigenin and acacetin. The conversion ratio = peak area of product / peak area of (product + substrate). The conversion ratio of positive protein SbCYP82D1.1 was calculated as a reference.

**Additional file 7: Figure S3. **Enzymatic test of microsomes with recombinant LpCYP82D-8 and -1 by UPLC and MS/MS. 4’OHWOR, 4’-hydroxywogonin.

**Additional file 8: Figure S4.** LpCYP82D-8 docking with apigenin (**a**), acacetin (**b**), 4’-hydroxywogonin (**c**), and hispidulin (**d**). The left chart is the overall docking with the substrate, the right chart is the binding domain of LpCYP82D-8 docking with the substrate. The molecule marked bright grey is the flavone substrate; the molecule marked green is ferriporphyrin.

**Additional file 9: Figure S5.** LpCYP82D-1 docking with apigenin (**a**), acacetin (**b**), 4’-hydroxywogonin (**c**), and hispidulin (**d**). The left chart is the overall docking with the substrate, the right chart is the binding domain of LpCYP82D-1 docking with the substrate. The molecule marked bright grey is the flavone substrate; the molecule marked green is ferriporphyrin.

**Additional file 10: Table S5. **FPKM of candidate LpCYP82D and LpUGT genes.

**Additional file 11: Table S6. **Locus and names of the CYP450 and UGT proteins in *Arabidopsis thaliana*.

**Additional file 12: Figure S6.** Identification of UGTs in *L. pauciflorus*. (**a**) Phylogenetic analysis of *L. pauciflorus* and *Arabidopsis* UGTs. The sequences were aligned using the ClustalW algorithm, based on the neighbor-joining method. The size of light blue represents the bootstrap value. (**b**) *L. pauciflorus* expression profiles. Hierarchical clustering for 187 LpUGTs was conducted based on transcript data.

**Additional file 13: Figure S7.** Characteristic of LpUGTs. (**a**) The motif structures of 13 selected LpUGTs. (**b**) SDS-PAGE analysis of the crude extract LpUGT95 and purified LpUGT95.

**Additional file 14: Table S7. **List of the represent functional CYP450 and UGT genes.

**Additional file 15: Table S8. **List of primers used in this study.

## Data Availability

GSA accession of all transcriptomic data is: CRA007588; GenBank number of genes used in paper as follow: LpCYP82D-8, OP096437; LpCYP82D-1, OP096438; LpUGT95, OP096439; LpUGT112, OP096440; LpUGT131, OP096441; LpUGT138, OP096442; LpUGT154, OP096443; LpUGT157, OP096444. LpUGT95 was suggested to be named as LpUGT71BF1 by the UGT Nomenclature Committee (https://labs.wsu.edu/ugt/, Dr Michael Court, michael.court@wsu.edu). All data generated or analyzed during this study are included in this published article and its Additional file.

## References

[1] Liu Y, Wagner H, Bauer R (1998). Phenylpropanoids and flavonoid glycosides from *Lysionotus pauciflorus*. Phytochemistry.

[2] Zhang YF, Shu ZD, Liu QM, Zhou Y, Zhang J, Liu H (2020). Nevadensin relieves food allergic responses and passive cutaneous anaphylaxis in mice through inhibiting the expression of c-Kit receptors. Food Funct.

[3] Müller L, Schütte LRF, Bücksteeg D, Alfke J, Uebel T, Esselen M (2021). Topoisomerase poisoning by the flavonoid nevadensin triggers DNA damage and apoptosis in human colon carcinoma HT29 cells. Arch Toxicol.

[4] Wang YQ, Weng ZM, Dou TY, Hou J, Wang DD, Ding LL (2018). Nevadensin is a naturally occurring selective inhibitor of human carboxylesterase 1. Int J Biol Macromol.

[5] Brahmachari G (2010). Nevadensin: Isolation, chemistry and bioactivity. Int J Green Pharm.

[6] Berim A, Gang DR (2013). The roles of a flavone-6-hydroxylase and 7-O-demethylation in the flavone biosynthetic network of sweet basil. J Biol Chem.

[7] Latunde-Dada AO, Cabello-Hurtado F, Czittrich N, Didierjean L, Schopfer C, Hertkorn N (2001). Flavonoid 6-hydroxylase from soybean (*Glycine max* L.), a novel plant P-450 monooxygenase. J Biol Chem..

[8] Zhao Q, Cui MY, Levsh O, Yang D, Liu J, Li J (2018). Two CYP82D enzymes function as flavone hydroxylases in the biosynthesis of root-specific 4'-deoxyflavones in *Scutellaria baicalensis*. Mol Plant.

[9] Berim A, Park JJ, Gang DR (2014). Unexpected roles for ancient proteins: flavone 8-hydroxylase in sweet basil trichomes is a Rieske-type. PAO-family oxygenase Plant J.

[10] Gao R, Lou Q, Hao L, Qi G, Tian Y, Pu X (2022). Comparative genomics reveal the convergent evolution of CYP82D and CYP706X members related to flavone biosynthesis in Lamiaceae and Asteraceae. Plant J.

[11] Cui L, Yao S, Dai X, Yin Q, Liu Y, Jiang X (2016). Identification of UDP-glycosyltransferases involved in the biosynthesis of astringent taste compounds in tea (*Camellia sinensis*). J Exp Bot.

[12] Cheng Y, Liu H, Tong X, Liu Z, Zhang X, Li D, Jiang X (2020). Identification and analysis of CYP450 and UGT supergene family members from the transcriptome of *Aralia elata* (Miq.) seem reveal candidate genes for triterpenoid saponin biosynthesis. BMC Plant Biol..

[13] Liu MH, Yang BR, Cheung WF, Yang KY, Zhou HF, Kwok JS (2015). Transcriptome analysis of leaves, roots, and flowers of *Panax notoginseng* identifies genes involved in ginsenoside and alkaloid biosynthesis. BMC Genomics.

[14] Yin Q, Han X, Han Z, Chen Q, Shi Y, Gao H (2020). Genome-wide analyses r a glucosyltransferase involved in rutin and emodin glucoside biosynthesis in tartaryz buckwheat. Food Chem.

[15] Yin Q, Han X, Chen J, Han Z, Shen L, Sun W (2021). Identification of specific glycosyltransferases involved in flavonol glucoside biosynthesis in ginseng using integrative metabolite profiles, DIA proteomics, and phylogenetic analysis. J Agric Food Chem.

[16] Yin Q, Wei Y, Han X, Chen J, Gao H, Sun W (2022). Unraveling the glycosylation of astringency compounds of horse chestnut *via* integrative sensory evaluation, flavonoid metabolism, differential transcriptome, and phylogenetic analysis. Front Plant Sci.

[17] Nelson DR (2018). Cytochrome P450 diversity in the tree of life. Biochim Biophys Acta Proteins Proteom.

[18] Berim A, Kim MJ, Gang DR (2015). Identification of a unique 2-oxoglutarate-dependent flavone 7-O-demethylase completes the elucidation of the lipophilic flavone network in basil. Plant Cell Physiol.

[19] Feng C, Wang J, Wu L, Kong H, Yang L, Feng C (2020). The genome of a cave plant, *Primulina huaijiensis*, provides insights into adaptation to limestone karst habitats. New Phytol.

[20] Xiao L, Yang G, Zhang L, Yang X, Zhao S, Ji Z (2015). The resurrection genome of *Boea hygrometrica*: a blueprint for survival of dehydration. Proc Natl Acad Sci U S A.

[21] Paddon CJ, Westfall PJ, Pitera DJ, Benjamin K, Fisher K, McPhee D (2013). High-level semi-synthetic production of the potent antimalarial artemisinin. Nature.

[22] Luo X, Reiter MA, d'Espaux L, Wong J, Denby CM, Lechner A (2019). Complete biosynthesis of cannabinoids and their unnatural analogues in yeast. Nature.

[23] Berim A, Gang DR (2018). Production of methoxylated flavonoids in yeast using ring A hydroxylases and flavonoid O-methyltransferases from sweet basil. Appl Microbiol Biotechnol.

[24] Trapnell C, Williams BA, Pertea G, Mortazavi A, Kwan G, van Baren MJ (2010). Transcript assembly and quantification by RNA-Seq reveals unannotated transcripts and isoform switching during cell differentiation. Nature Biotechnol.

[25] Li B, Dewey CN (2011). RSEM: accurate transcript quantification from RNA-Seq data with or without a reference genome. BMC Bioinformatics.

[26] Love MI, Huber W, Anders S (2014). Moderated estimation of fold change and dispersion for RNA-seq data with DESeq2. Genome Biol.

[27] El-Gebali S, Mistry J, Bateman A, Eddy SR, Luciani A, Potter SC (2019). The Pfam protein families database in 2019. Nucleic Acids Res.

[28] Tamura K, Stecher G, Kumar S (2021). MEGA11: molecular evolutionary genetics analysis version 11. Mol Biol Evol.

[29] Pompon D, Louerat B, Bronine A, Urban P (1996). Yeast expression of animal and plant P450s in optimized redox environments. Methods Enzymol.

[30] Truan G, Cullin C, Reisdorf P, Urban P, Pompon D (1993). Enhanced in vivo monooxygenase activities of mammalian P450s in engineered yeast cells producing high levels of NADPH-P450 reductase and human cytochrome b5. Gene.

[31] Bradford MM (1976). A rapid and sensitive method for the quantitation of microgram quantities of protein utilizing the principle of protein-dye binding. Anal Biochem.

[32] Trott O, Olson AJ (2010). AutoDock Vina: improving the speed and accuracy of docking with a new scoring function, efficient optimization, and multithreading. J Comput Chem.

