# Supplementary figures and images for: Integrated multi-omics analysis and microbial recombinant protein system reveal hydroxylation and glycosylation involving nevadensin biosynthesis in Lysionotus pauciflorus
Source: Microb Cell Fact. 2022 Sep 19;21:195. doi: 10.1186/s12934-022-01921-2 (PMC9484059; doi:10.1186/s12934-022-01921-2)

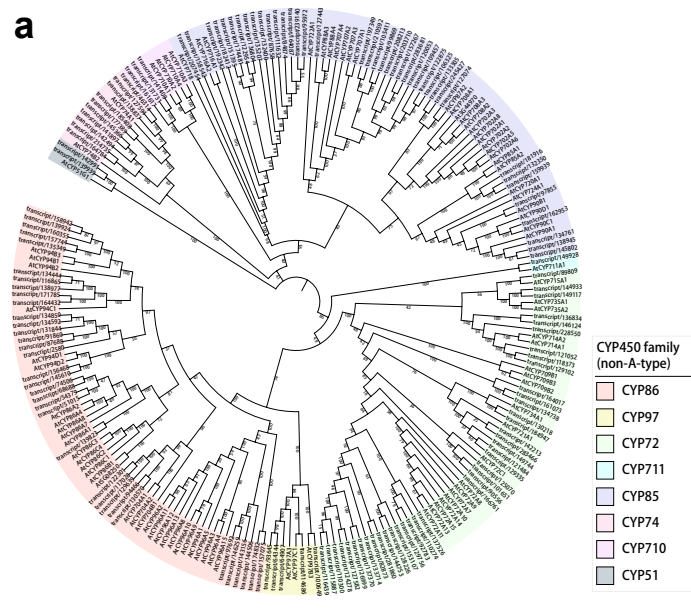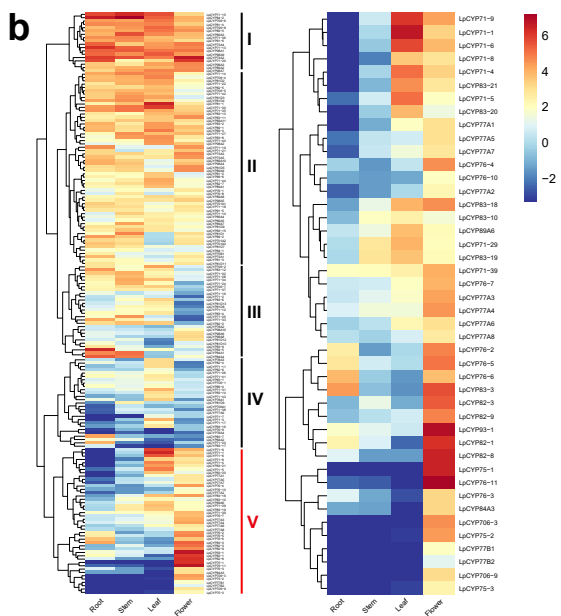

Supplement: Supplementary file 4 — Additional file 4: Figure S1. Identification of CYP450s in L. pauciflorus. (a) Phylogenetic evolutionary analysis of non-A-type CYP450s from L. pauciflorus. (b) Hierarchical clustering for expression profiles of 175 A-type LpCYP450s. [file 12934_2022_1921_MOESM4_ESM.pdf]

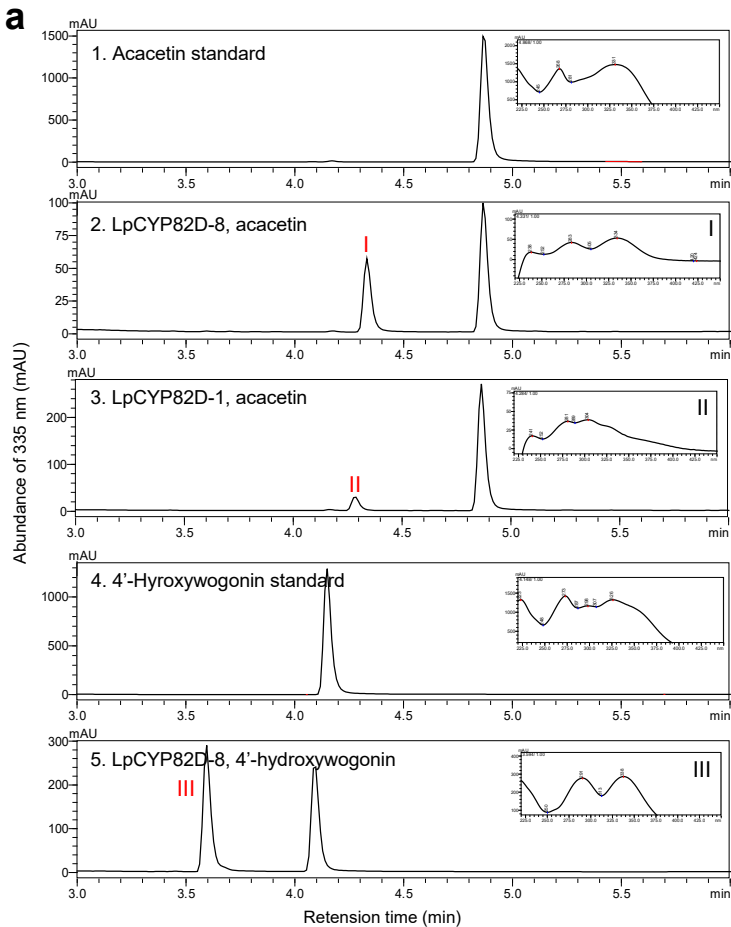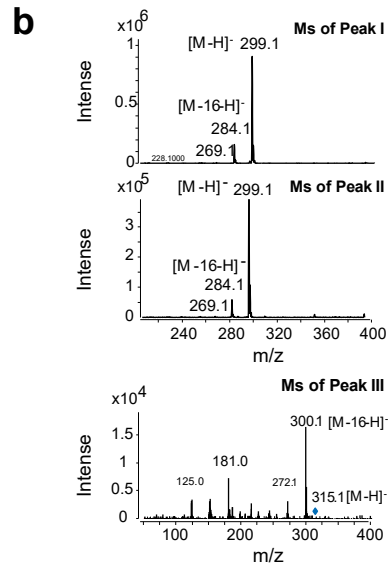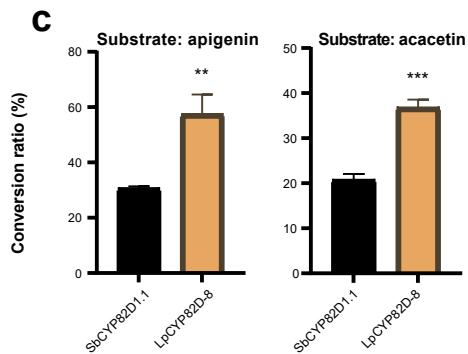

Supplement: Supplementary file 6 — Additional file 6: Figure S2. The identification of LpCYP82D-8 and -1 function in vivo by UPLC and MS/MS, and the conversion ratio of LpCYP82D-8. (a) UPLC profile of LpCYP82D-8 and -1 treated with different substrates. (b) MS/MS results of yeast expressing LpCYP82D-8 and -1 added acacetin standard as substrate. (c) The conversion ratio of LpCYP82D-8 treated with apigenin and acacetin. The conversion ratio = peak area of product / peak area of (product + substrate). The conversion ratio of positive protein SbCYP82D1.1 was calculated as a reference. [file 12934_2022_1921_MOESM6_ESM.pdf]

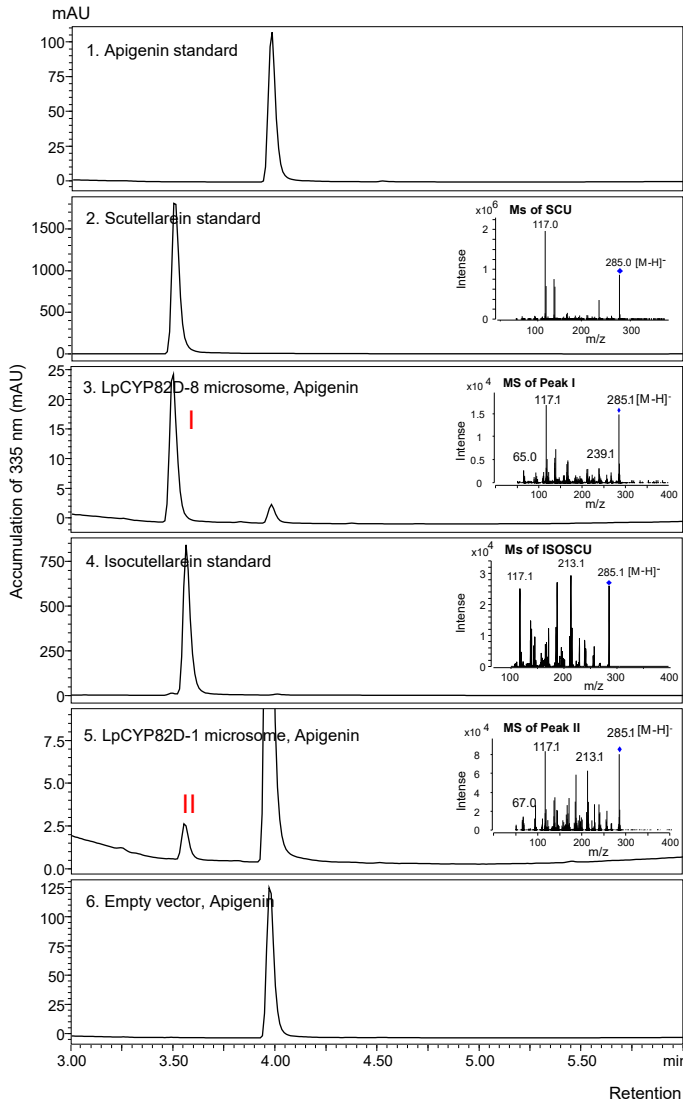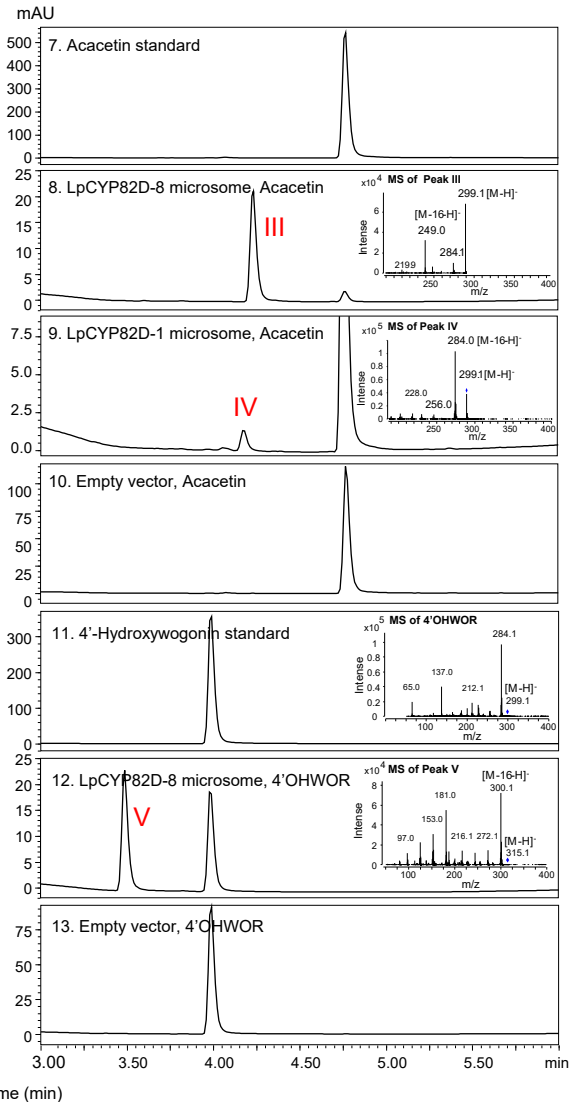

Supplement: Supplementary file 7 — Additional file 7: Figure S3. Enzymatic test of microsomes with recombinant LpCYP82D-8 and -1 by UPLC and MS/MS. 4’OHWOR, 4’-hydroxywogonin. [file 12934_2022_1921_MOESM7_ESM.pdf]

**a**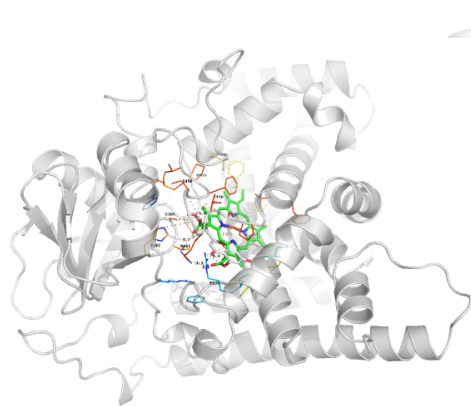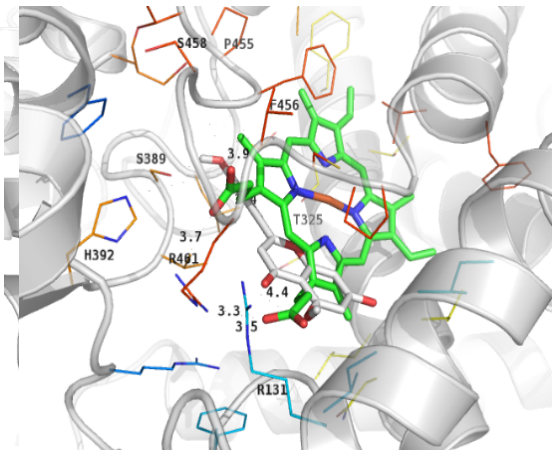**b**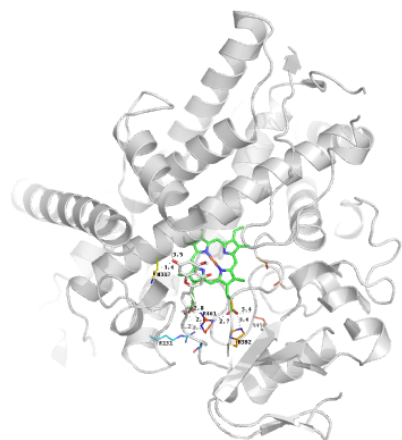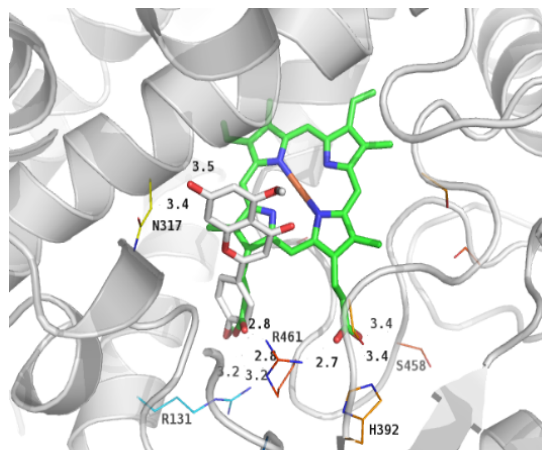**c**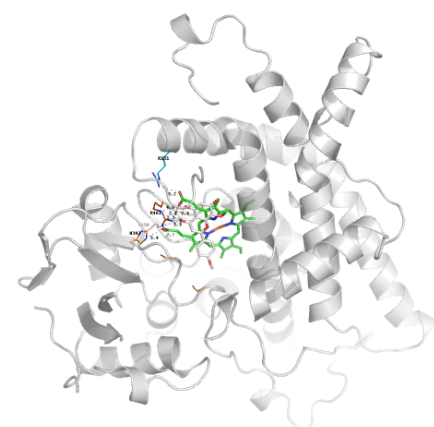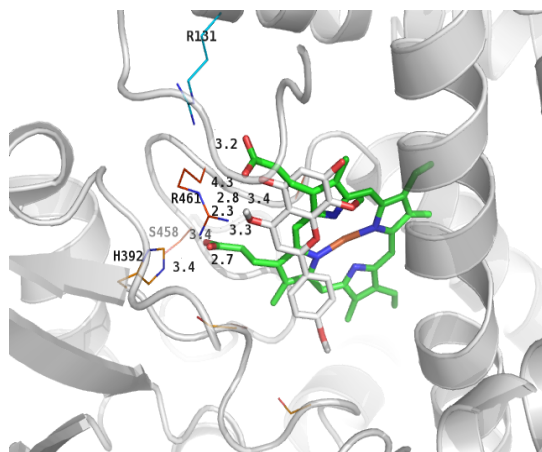**d**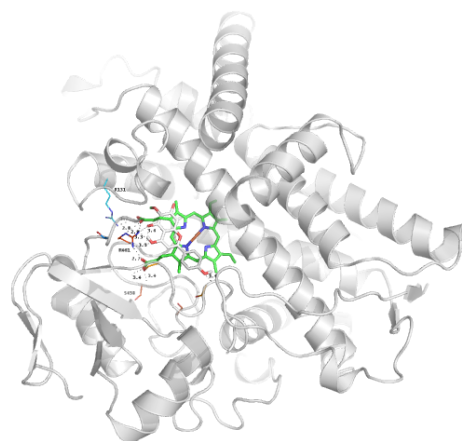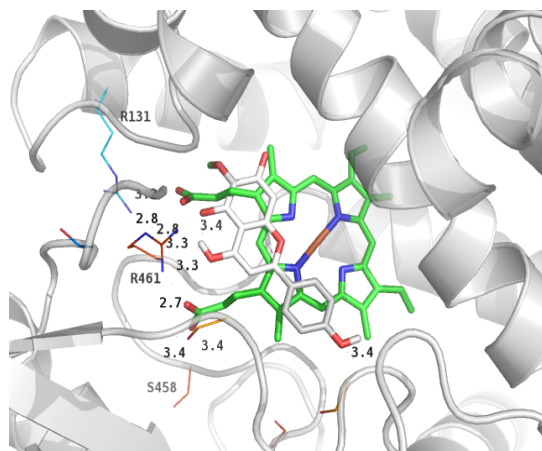

Supplement: Supplementary file 8 — Additional file 8: Figure S4. LpCYP82D-8 docking with apigenin (a), acacetin (b), 4’-hydroxywogonin (c), and hispidulin (d). The left chart is the overall docking with the substrate, the right chart is the binding domain of LpCYP82D-8 docking with the substrate. The molecule marked bright grey is the flavone substrate; the molecule marked green is ferriporphyrin. [file 12934_2022_1921_MOESM8_ESM.pdf]

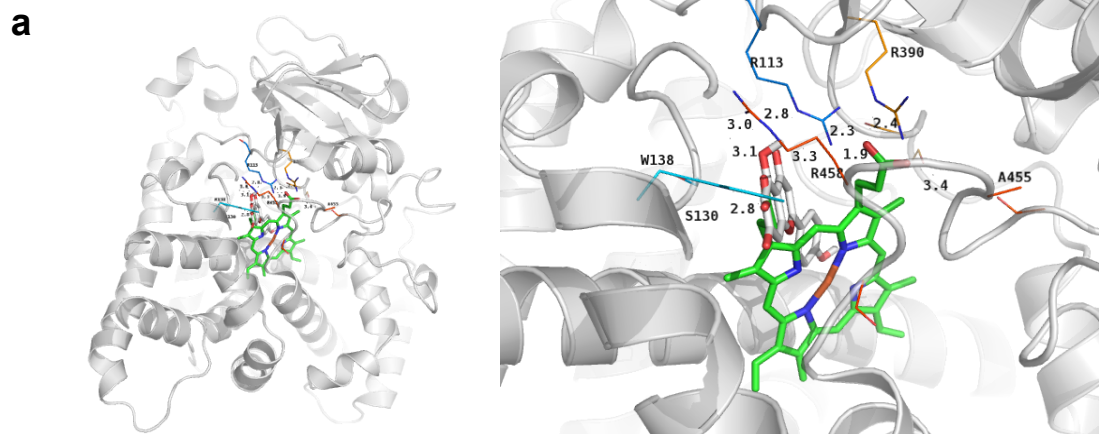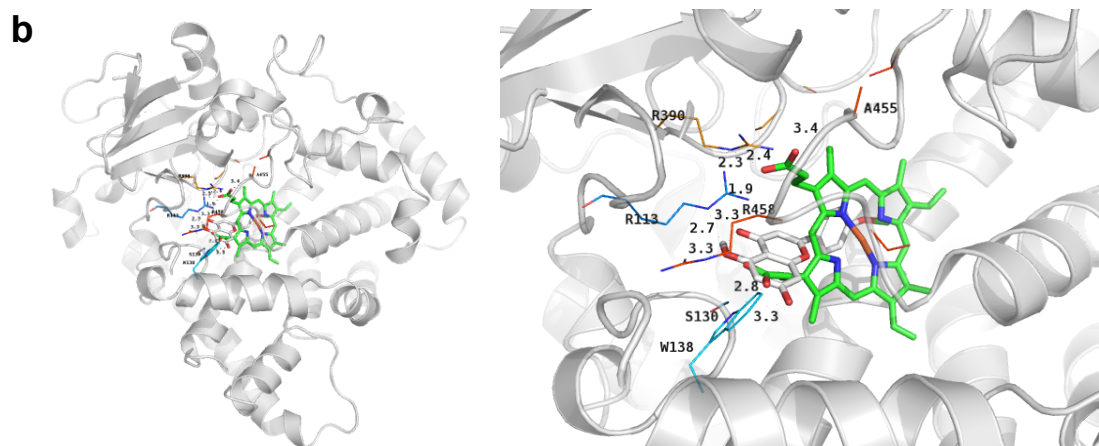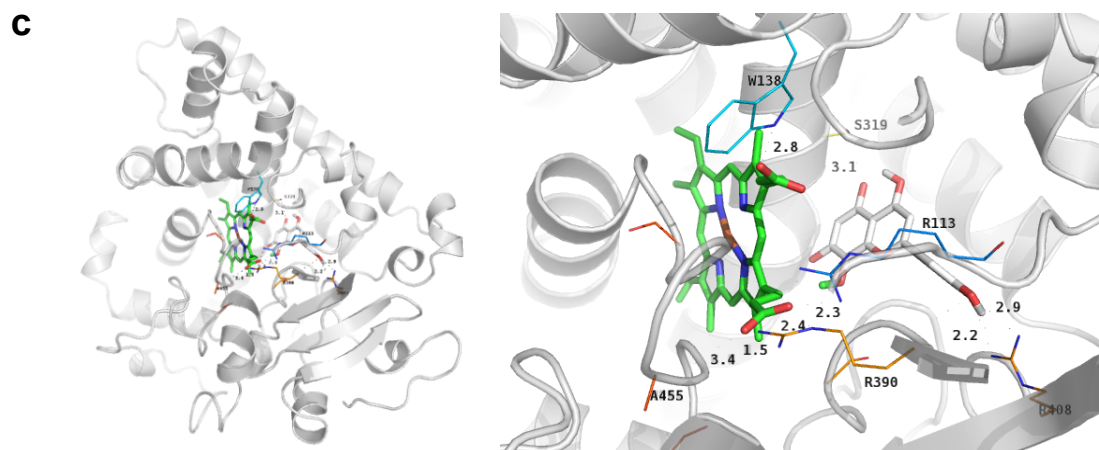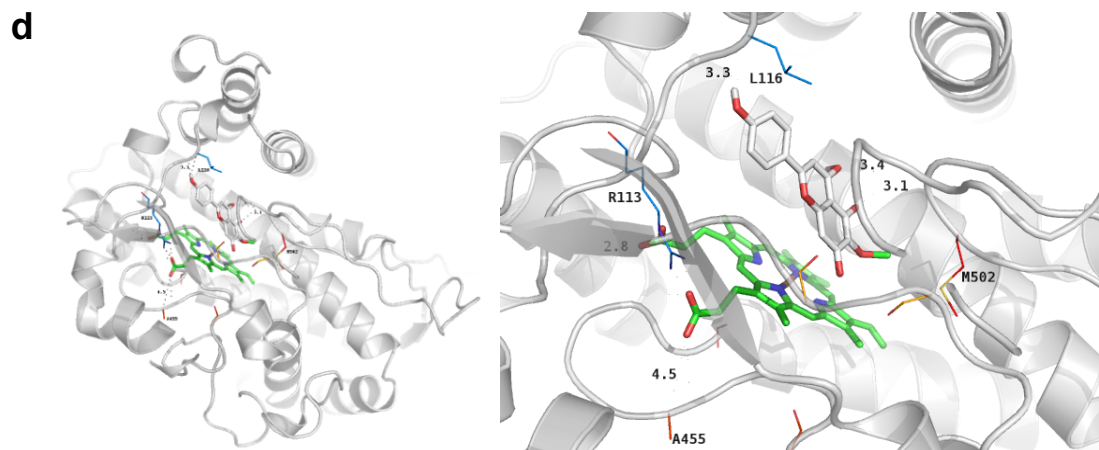

Supplement: Supplementary file 9 — Additional file 9: Figure S5. LpCYP82D-1 docking with apigenin (a), acacetin (b), 4’-hydroxywogonin (c), and hispidulin (d). The left chart is the overall docking with the substrate, the right chart is the binding domain of LpCYP82D-1 docking with the substrate. The molecule marked bright grey is the flavone substrate; the molecule marked green is ferriporphyrin. [file 12934_2022_1921_MOESM9_ESM.pdf]

**a**

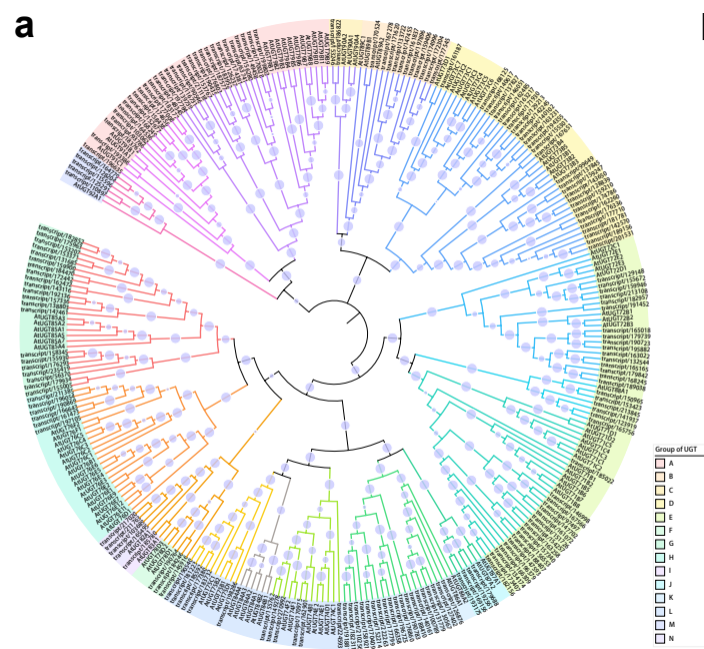

**b**

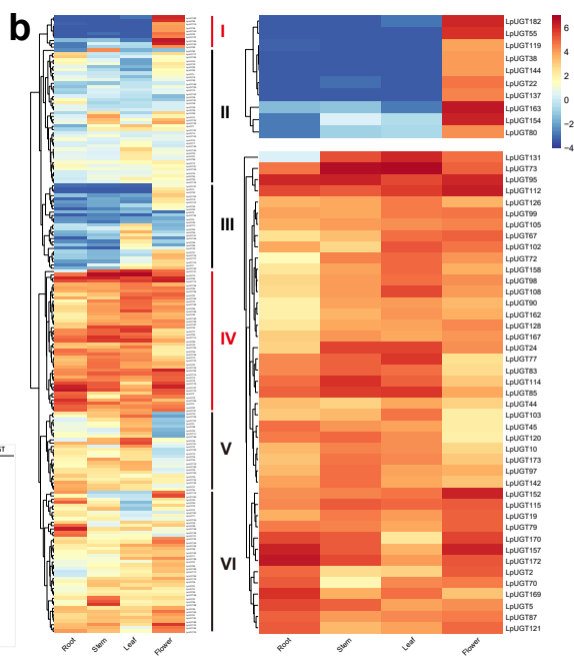

Supplement: Supplementary file 12 — Additional file 12: Figure S6. Identification of UGTs in L. pauciflorus. (a) Phylogenetic analysis of L. pauciflorus and Arabidopsis UGTs. The sequences were aligned using the ClustalW algorithm, based on the neighbor-joining method. The size of light blue represents the bootstrap value. (b) L. pauciflorus expression profiles. Hierarchical clustering for 187 LpUGTs was conducted based on transcript data. [file 12934_2022_1921_MOESM12_ESM.pdf]
