# Supplementary material for: Integrated multi-omics analysis and microbial recombinant protein system reveal hydroxylation and glycosylation involving nevadensin biosynthesis in Lysionotus pauciflorus
Source: Microb Cell Fact. 2022 Sep 19;21:195. doi: 10.1186/s12934-022-01921-2 (PMC9484059; doi:10.1186/s12934-022-01921-2)
Supplement: Supplementary file 13 — Additional file 13: Figure S7. Characteristic of LpUGTs. (a) The motif structures of 13 selected LpUGTs. (b) SDS-PAGE analysis of the crude extract LpUGT95 and purified LpUGT95. [file 12934_2022_1921_MOESM13_ESM.pdf]

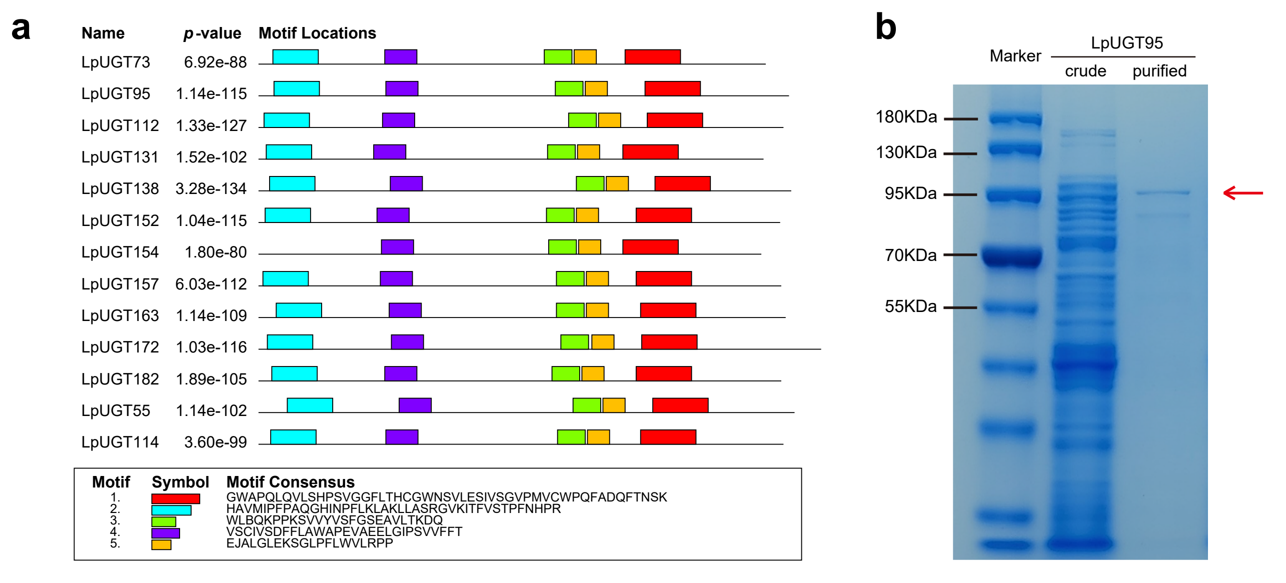
a
Name
p-value
Motif Locations
LpUGT73
6.92e-88
LpUGT95
1.14e-115
LpUGT112
1.33e-127
LpUGT131
1.52e-102
LpUGT138
3.28e-134
LpUGT152
1.04e-115
LpUGT154
1.80e-80
LpUGT157
6.03e-112
LpUGT163
1.14e-109
LpUGT172
1.03e-116
LpUGT182
1.89e-105
LpUGT55
1.14e-102
LpUGT114
3.60e-99
Motif
Symbol
Motif Consensus
1. GWAPQLQVLSHPSVGGFLTHCGWNSVLESIVSGVPMVCWPQFADQFTNSK
2. HAVMIPFPAQGHINPFLKLAKLLASRGVKITFVSTPFNHPR
3. WLBQKPPKSVVYVSFGSEAVLTKDQ
4. VSCIVSDFFLAWAPEVAEELGIPSVVFFT
5. EJALGLEKSGLPFLWVLRPP
b
Marker
LpUGT95
crude
purified
180KDa
130KDa
95KDa
70KDa
55KDa
